# Trends in the Study of Cultural-Historical Phenomena on the Internet (based on a study of Russians’ attitudes towards money)

**DOI:** 10.11621/pir.2022.0107

**Published:** 2022-03-30

**Authors:** Anatoly L. Zhuravlev, Yury P. Zinchenko, Dzhuletta A. Kitova

**Affiliations:** a Institute of Psychology of the Russian Academy of Sciences, Moscow, Russia; b Lomonosov Moscow State University, Moscow, Russia; c Psychological Institute of the Russian Academy of Education, Moscow, Russia

**Keywords:** Attitude toward money, social networks, information technologies, ideas about income and expenses, attitude toward time, expert analysis.

## Abstract

**Background:**

The development of information technologies has led to the intensification of sociocultural interaction, allowed the creation of new systems for storing and processing information, and provided space for users to share their opinions, ideas, and standpoints. Thus, the Internet has become a major social-humanitarian scientific space. In this modern scientific space, one can single out a wide range of studies in psychology that show which topics are most popular and most widely discussed, or which moral grounds the participants of radical political movements share. Such studies show, for example, that U.S. working people experience psycho-physiological strain, and that infectious diseases spread more easily under modern conditions.

**Objective:**

This study focused on the attitude that users of the Twitter social network hold towards money.

**Design:**

It was carried out by analyzing the texts of messages posted by Russian and Japanese users (background research) which contained the word “money.” The research methods included program tools for word frequency analysis, semantic grouping of content, and analyzing the emotional nature of informal short messages. To interpret the results, the authors used expert analysis, theoretical justification, and content analysis.

**Results:**

We found that Russians’ attitudes toward money can be divided into eight main categories: people, time, country, expenses, economy, philosophical speculations, power, and income. The main economic concerns were centered on the expenses and income coming from salaried jobs. Russians’ major expenses were mainly associated with everyday financial problems. A comparison of Russian and Japanese messages revealed a number of clear-cut psychological differences.

**Conclusion:**

In conclusion, we point out that analyzing “digital traces” helps uncover a variety of psychological factors influencing human life and behavior. Within the framework of this kind of study, it seems very promising to single out the interconnection between the population’s overall psychological features and a given society’s existing social-economic circumstances.

## Introduction

The development of information technologies is becoming a crucial factor for transforming modern society. It determines the arrival of new forms of interaction between authorities, social institutions, business, and the population. From the *social-psychological* standpoint, such a development leads to transformations of group values, norms of behavior, and social roles. Among the typical characteristics of this process are the considerable widening of sociocultural interaction, the large-scale accumulation of varied interests among different cultures and ethnic groups, and the emergence of new spaces for young people’s socialization. The existing systemic autonomy of the Internet space, and the absence of rigid external control and regulation of it, allows one to consider it a new, developing, and relatively independent *cultural subsystem*.

The importance of the Internet is determined not only by its procedural ability to change the communicative milieu and form a new social space, but also by involving huge numbers of people in this process. By the year 2021 4.66 billion people were Internet users (All Internet statistics …, 2020). In this respect it is also important to note that the child audience of the Internet is constantly growing, which has immediate consequences for how human culture is evolving; its mentality is “directly affected” (Pelipenko, 2015, p. 163). For example, it has been shown that, on average, *pre-school* (!) children use various types of electronic gadgets and spend 13.42 hours a week in virtual space (Funk et al., 2009).

From the standpoint of *information*, the integration of means of communication with computer networks helps to build powerful systems for storing huge amounts of data (and processing these data), while the fact of being connected to the Internet’s international network provides additional opportunities for getting into the worldwide community’s information space. Thus, one gains access to information sources in almost every part of the world.

The existing informational milieu provides people with new platforms for communication: sites, forums, blogs, online diaries, thematic communities, and so on. Among other platforms, the most popular ones are social networks: their audience by 2021 has surpassed the 4.2 billion mark, which is 53.6% of the world population (All Internet statistics …, 2020). The Internet creates new opportunities for people’s activity by allowing users to generate their own information and spread their worldview without any special preparation.

From the *scientific* point of view, the Internet reflects the on-going process of broadcasting opinions, ideas, standpoints, attitudes, and worldviews typical of both individuals and social groups. The networks also collect personal data in both their subjective form (opinions expressed, for instance) and their objective format (login coordinates, contacts, profiles, statuses, etc.) The information obtained in this way provides broad opportunities for studying innumerable objective and subjective *characteristics* of people and social groups functioning on the Internet, which makes it a big social-humanitarian field for study (digital trace studies are no exception). By way of example, according to E.R. Agadullina’s survey, the connections between certain psychological characteristics (like the Big Five factors) and “digital traces” are most reliably established when the latter are collected directly from people’s profiles, and not from what the respondents answer in a questionnaire (Agadullina, 2015).

### State of the research using information technologies

In the modern study space, the technologies used for analyzing “digital traces” are connected with processing the users’ objective characteristics (gender, age, geography, frequency of use of information resources, and so forth), as well as with singling out people’s subjective features (groups, societies). This kind of information cannot be obtained *directly* (for example, simply by counting certain positions and their subsequent statistical processing), and to extract it, one needs complex *program products* and well-founded *theoretical grounds* (one needs to address the already proven, conventional scientific conceptions, laws, or facts) that would constitute the basis to explain it. Below we provide a review of such research, in order to analyze the state of affairs within this trend in modern psychological studies.

One can identify quite a number of studies within different *branches of psychological science*:

- *the psychology of economics*: a complex analysis of articles (João et al., 2016) on the problem of C-RM marketing (the research analyzed 246 articles in 40 journals dating from 1988 to 2013) revealed the topics which were most widely discussed (“brand compliance,” “law and ethics,” “corporate and social identification”), as well as those topics which were rapidly gaining popularity (“social taboo,” “moral debate”);
- *legal psychology*: A study of the moral attractiveness of extremism in the United States, performed by analyzing 5000 messages by representatives of far-right political movements (alt-right) and 5000 ones by representatives of the extreme left wing (Antifa), revealed differences only as to the principle of internal group loyalty, while no evidence of high moral grounds was found in either of the groups (Alizadeh, et al., 2019);
- *the psychology of management*: An analysis of the dynamics of Americans’ emotional state during the week was performed in order to study the “effect of recovery from work” on weekends. It showed that work-related elements have well-pronounced *negative* load throughout the week, but also on the weekends: the results obviously point to the “cumulative” strained psycho-physiological state of U.S. working people (Wang, et al., 2016);
- *the psychology of professional communities*: A comparative analysis of messages from readers of journals on psychology and political sciences was carried out by studying articles from Web of Science and Altmetric.com and discussion of them on the Twitter social network. The study analyzed 91,826 tweets on psychology-related problems and 29,958 ones on political science issues. It showed that the messages concerning the publications in psychological journals gave rise to a much greater number of discussions on a wider range of subjects than those on political sciences (Zhou & Na, 2016);
- *the psychology of mass media*: A study of changes in the perception of AIDS, performed on the basis of 446 articles in two Italian newspapers (*La Repubblica* and *Corriere della Sera*) published between 1985 and 1990, and between 2005 and 2010, singled out five thematic clusters of opinion (medical help, support from family, science and religion discourse, social avoidance/ isolation, and health policy) and showed that in the latter period, the attitude toward this illness was more tolerant (Caputo, et al., 2016);
- *the psychology of art*: a study of the impressions and psychological effects that people with different cultural backgrounds experience while watching stage performances and dances of Japan, China, and South Korea showed that traditional arts are perceived differently, depending on the ethnic and cultural background of the spectators (the study was performed by way of automatic analysis of responses to an open-question survey (Zheng, et al., 2020).

### Theoretical-methodological approaches and study methods

From the theoretical-methodological point of view, people’s attitudes towards social phenomena are highly abstract concepts that reveal both the *social* context of the issue (dependance on historical and cultural features of the milieu one lives in, etc.) and one’s *individual personality* traits in relation to one’s life circumstances. Another feature of the phenomenon of “attitude” is the ability to concretize it by specifying its *structural* and *semantic*, as well as *cognitive*, *emotional*, and *motivational-behavioral*, components.

From the practical point of view, the phenomenon of attitude also has specific features and advantages. Firstly, an attitude forms the basis for people’s *views of the world*, *activity,* and *behavior* and largely determines them. Secondly, an attitude can be easily identified through studying an individual’s speech according to the following scheme: content — structure — cognition — emotions — motivation. Thirdly, it is also interesting to try to identify a person’s general *emotional* attitude toward various phenomena of external life and toward himself through analyzing his psychological evaluations of an activity (Zhuravlev & Kitova, 2020b). All these aspects are related to the fact that the phenomenon of attitude is an extremely *popular and widespread* object of research in various branches of psychology.

The object of the present study — *attitude toward money* — represents research in both the field of *attitude phenomena* and in that of *economic phenomena*, generally. Bearing in mind all the above-mentioned aspects of the problem, it comes as *no* surprise that the analysis of the population’s attitude toward money can reveal certain psychological features characteristic of a great number of people, features that can be seen as *elements* of the Russian *mentality*^1^. This is our first theoretical assumption. The second one is related to the fact that a person’s attitude toward money is viewed as a “conscientious and subjective-selective idea of money” (Deineka, 2000, p. 79), manifesting itself both in the person’s handling of money and his ideas about it.

Thus, the *objective* of our research was to study the macroeconomic (that is, relevant to the society on the whole) features of Russians’ attitudes toward money.

## Method

The *object* of the research was a group of users of the Twitter social network, and its *material* consisted of the content of text messages that included the word “money,” and had to do with all kinds of life situations in which the users founnd themselves, without any exceptions or limitations. The messages were collected using the continuous sampling method during the month between June 11 and July 11, 2021. They were processed after eliminating advertisements and recurrent text material/bots; the material was then automatically divided into tokens (morphological stemming was performed with the help of user vocabularies); then the tokens were grouped and subsequently analyzed from the viewpoint of semantics, frequency, and the emotional load of words and messages.

A total of 3984 messages were collected and processed. In line with the background study, it was suggested that we use the corresponding (identical) methods to reveal the specific features in the attitude towards money of the Japanese, as Japan is a country with a very specific ethnocultural history of economy and its development, which differs greatly from that of Russia.

### Procedure

The *automatic* methods used within the study included program instruments for assessing *word frequency*, grouping *contents* by *semantic* criteria, and analyzing the *emotional* tone of informal short messages; these instruments were elaborated by M. Kitov (for more information on the methods used, see *Psychological studies in the Internet space* …, 2020). To *interpret* the results obtained, we used supplementary methods such as *expert analysis*, method of *theoretical justification*, and *content analysis*. All the clarifications concerning the use of concrete techniques of *qualitative* analysis were made along with discussion of the research results.

## Results

### Empirical analysis of the Twitter users’ attitude towards money

Our *first step* was to semantically segregate all the text messages, which allowed us to single out eight main subgroups. They are presented in *Table 1*, ordered from the most cited ones (the most recurrent in the whole bulk of messages) to the least. The *leading* position was occupied by *earning money* to provide for oneself, which connects money and the social world. The dominant social category with which the idea of earning money was most often connected was that of *children* (a family’s income is mostly spent on children, and it is for children that the state is supposed to care to a greater extent) and their parents, for whom much public empathy is reserved, given the heavy expenses they carry while raising children and providing for their care.

**Table 1:** Frequency characteristics of words in generalized semantic categories

Semantic categories	Frequency of words from different categories
PEOPLE Example: … *money* is not the most important thing; it is much more important that you have your nearest and dearest by your side, that you feel support, positive vibes and have memorable moments in your life.	people — 165, children — 108, person —63, mothers — 46, parents — 34, school children — 34, etc.
TIME Example: This year it’s time for me to go to university but I have no idea which one to choose. I am mortally afraid of choosing the wrong one and just throwing away time and *money*.	year — 79, month — 67, time — 67, years — 54, day — 45, moment — 28, week — 26, hour — 23, etc.
COUNTRY Example: A country must invest *money* in its people.	Russia — 83, country — 56, (state) budget — 41, people — 93, development — 30, roads — 28, (national) projects — 28, etc.
EXPENSES Example: … if you decide to take a mortgage on your flat, you need to have enough *money* for the initial payment plus 20-25 thousand rubles for other deal-related expenses.	home — 59, family — 51, flat — 45, things — 33, renovation — 25, debts — 30, gas — 24, shop — 24, ticket (travelling and leisure) — 26, etc.
ECONOMY Example: The Russian economy is like this: bankers take out loans at low interest rates in the West and process this *money* in Russia, lending it to the local people on high rates.	rubles — 72, cards — 65, accounts –49, taxes — 46, banks –29, business –26, etc.
PHILOSOPHY OF LIFE Example: Your child must be your pride. You wife must be your honor. Your husband must be your support. Your friends must be loyal. *Money* must be superfluous. Health must be strong. And life must be beautiful!	life — 61, questions — 59, rights — 33, words — 27, sense — 26, USA — 29, problems –26, etc.
POWER Example: Neither *money* nor power nor popularity as such can spoil a person.	civil servants — 31, missive (of the presidents) — 31, power — 137, elections — 25, etc.
INCOME Example: I believe that people’s real income reveals itself in the possibility of spending *money* on holidays.	work — 94, salaries — 30, payments — 24, etc.

The interconnection between money and *time* was the second most frequently cited subgroup. This was not connected with the necessity to earn money (and fill one’s time with activities aimed at improving one’s standard of living): it rather consisted of the fact that as time passed, the users’ financial situation did not improve correspondingly, thus leaving the users’ income expectations unrealized.

The third category in importance was the *state* (not the population itself), which was still expected to provide a high level of income and use all the resources at its disposal to guarantee the overall (financial included) well-being of the people.

*Expenses* (the fourth most important group) were discussed by the users in the context of individuals’ daily routines. This category intersected the first one (people —children — parents), since all the expenses discussed by the users were connected with the need to support the family financially, bring up the children, and maintain the family’s home. A separate point was the need to pay for the housing and communal services that the users considered too expensive (this issue becomes more pressing as gas supply prices for households grow).

By way of illustration, we cite a message that, while not typical or common to many users, was still quite indicative of people’s attitude toward communal services and their costs: “… I feel sad because now the financial situation of our family is really bad, and, most likely, I’ll have to spend all my money on paying the bills: four of the five members of my family do not earn any money at all.” The only category that was not connected with *everyday* expenses was holidays, which were connected with a category such as “tickets.” This category was usually associated with visiting mass cultural events and travelling for recreation or pleasure.

The fifth most cited category — *economy* — was represented in the messages through discussions of the state’s financial, credit, and fiscal systems; the texts said little about production or the private sector of the economy. The same outlook was evident in how the users analyzed economic policies *philosophically*: their statements concerned the external circumstances and conditions they lived in, rather than the need to undertake something themselves and change their lives. This subject was in the sixth position.

The external locus of control in assessing financial problems was most visible in statements about power (position seven). In these statements the users most frequently criticized civil servants who, in their opinion, are to *blame* for the Russians’ poor living conditions by inhibiting the development of the economy (the most recurrent word in this context was “to rob”), failing to perform their direct duties, and being unable to fulfill the commands of the higher authorities (among which were the President’s stated mission).

The ideas concerning *income* (position eight) in the users’ messages were most frequently related to salaried jobs, and were mainly limited to waiting for a salary, disbursements, or payouts, rather than connected in any way with individual personal strategies of earning money or with the level/nature of economic activity. As an extreme case of such an attitude, we can cite the following message, although reservations should be made given the author’s sarcastic and ironical tone: “… Why can’t I just stay at home, get *money* for it, and spend it on BTS merch? Life is pain…”

The subgroups described above do not comprise all the categories that can be found in the users’ statements. Examples of other attitudes are shown in the following low-frequency statements concerning the activities of various groups of people:

- *psychologists* — “a practicing psychologist is a person who gets *money* from people for sorting out all their mess consisting of home, career, and mental problems;”
- *scientists* — “Russian science is a bottomless pit. Almost all the *money* the government invests in it is spent on the astronomical salaries for a whole army of academicians. No other country in the world has as many members of academies as Russia… But there is a very small number of scientists in Russia;”
- *wealthy people* — “How can normal people have loads of *money*? The answer to this question gives rise to the conclusion: the quantity of rich people is like a list of madmen.”

It is important to bear in mind that in the semantic groups specified within this study, we do not have singular examples of statements (which are innumerable on the Internet), but the truly widespread concepts shared by a great number of Russians. As an example of a *typical* such attitude, we can provide a phrase demonstrating how the majority of users mistrusts civil servants: “If only the local bureaucrats could be trained to react properly when they have *money* at their disposal: they must use it to the people’s benefit, and not to raise their own salaries and remunerations.”

The *second stage* of the study presupposed applying the inductive-deductive strategy to analyze the sample (messages) according to the specific theoretical approaches known from other sources (grounded theory)^2^. We analyzed the messages about the users’ financial problems and their *expenses* and *incomes*: their incomes were considered with emphasis on their *sources*, and their expenses were grouped into those connected with *consumption* and *accumulation* (savings and investments). The positions specified according to this principle were coded correspondingly (incomes/ expenses), and subsequently counted.

**Figure 1.**
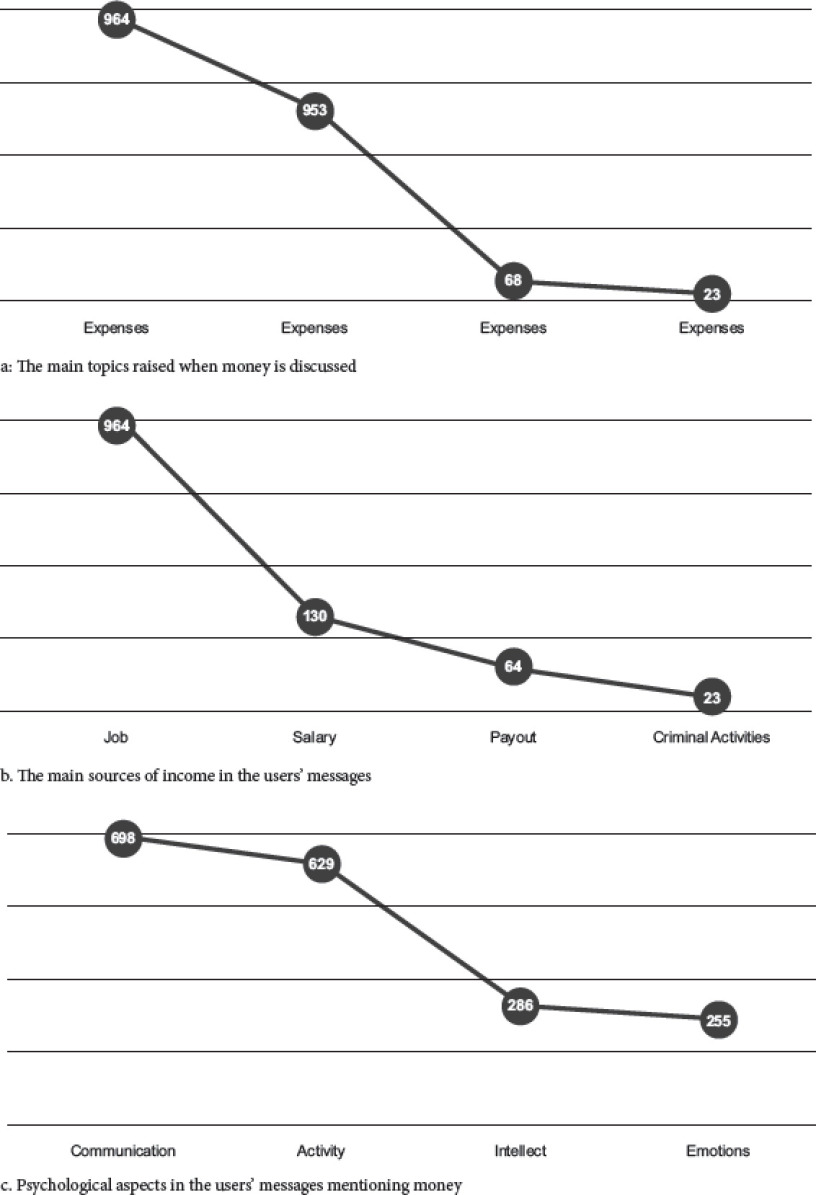
The Twitter users’ attitudes toward money: specific features (number)

As *Figure 1a* shows, the messages dealing with expenses outnumbered those discussing incomes, while savings and investments were rarely discussed or considered by the users.

The next stage of coding involved analyzing the messages on income sources (*Figure 1b*). As became evident, the structure of incomes most often allowed us to identify their source as the public sector (job, salary, or payout), while really high incomes were associated with criminal activities, which are regarded as the most efficient way of becoming rich. This last idea needs more detailed analysis if we want to learn on what grounds such assumptions are made.

If we take into account the psychological characteristics of the attitudes toward money represented in the users’ messages, we find that people mostly mentioned the function of *social exchange* (communication, mutual help, and support); they also understood that earning money required a certain *intellectual background* and competences, as well as concrete skills and abilities making oneself suitable for a particular kind of activity (labor, job qualifications, etc.) — see *Figure 1c*. In their messages the users also mentioned their emotional states connected with having or not having money.

If we rely on the trend we have identified, — namely, that the users regarded the circulation of money in the light of mutual help and social exchange — then it would be interesting to take a closer look at the intention of some users to give up this position (probably because of its “counterproductive” nature). One of the messages went as follows: “One shouldn’t spend his life on other people and thus throw away *money* and resources. It is necessary to become an EGOIST!” This position, although it sounds like a praise of egoism as a philosophy of life, rather reflects the opposite values of the author: *special efforts* are needed to reject them. Thus, being socially oriented can be regarded as the leading *psychological factor* of Russian users’ attitudes toward money.

At the *third stage* of the study, we analyzed the *emotional* tone of the messages. Our analysis revealed that the emotional character of the Russians’ attitude towards money was unipolar and exclusively negative: no positive or even neutral emotional evaluations related to money were found in the users’ messages. Below there are a few examples that illustrate this point: the intensity of negative evaluation grows from one sentence to another (*Table 2*).

**Table 2:** Examples of messages with negative emotional background

Emotional tone	Example of messages (tweets)
Examples of mes- sages with *negative* emotional back- ground	… It’s quite an ordinary job for which quite ordinary *money* is paid. … God, I do need *money* but I don’t have the least idea of how to earn it. … Auction means very big *money* and a very shadowy kind of business. … Make children’s sport and hobby clubs free of charge so that all the children could benefit, not only those whose parents have *money*. … Why does a child have no mention of a father in his documents? The answer is, to get payouts! They don’t work, they don’t pay taxes, they just spend the children’s *money* on booze…

**Figure 2.**
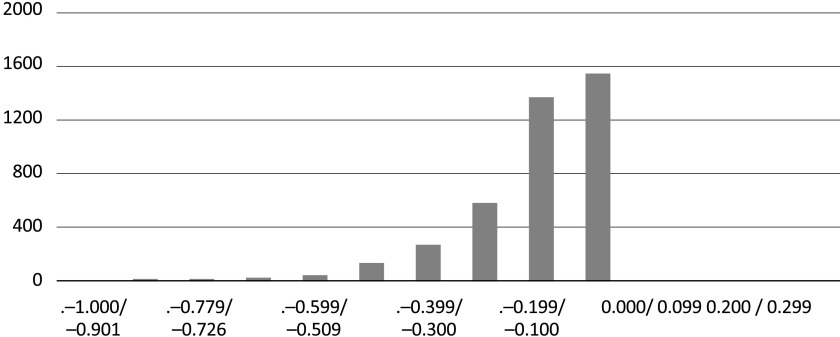
Distribution of emotional tone in the users’ messages (June-July 2021) (thousands).

For convenience purposes, we divided the characteristic features of the emotional spectrum expressed revealed within the study into 20 parts (*Figure 2*). The figure shows that the most widespread modalities were: slightly negative (1543); moderately negative (1363); expressly negative (586); sharply negative (277); extremely negative (133); expressive vocabulary (48); and obscene vocabulary (29).

If our starting point is the idea shared by many researchers who have studied attitudes towards money, — namely, that this attitude as an economic-psychological phenomenon “accompanies social exchange and is able to produce a compensatory therapeutic effect” (Deineka, 2000, p. 79) — then the Russians’ general psychological state with respect to money can be defined as *unsatisfied*, which means that the state of society as a whole can be characterized as tense.

**Figure 3.**
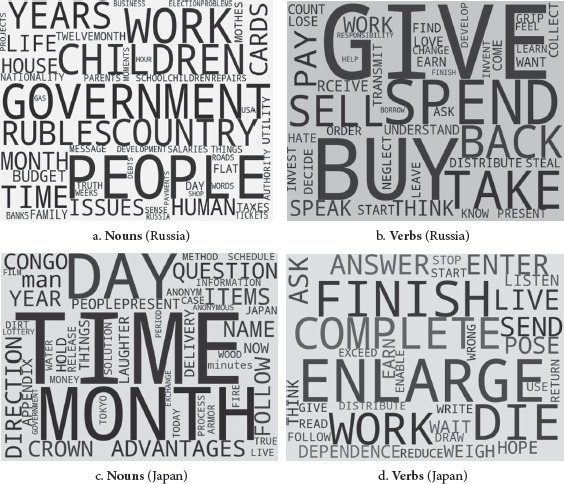
Psychological features of attitude towards money in Russia and in Japan

The *fourth stage* of the study involved frequency analysis of the words used in the whole sample, and subsequent comparison of the results with the corresponding data from the Japanese users. To give an overall picture, we specified the 50 most popular words, which are presented below as a weighted list (*Figure 3*). The words are represented by singular categories (without linking elements) and the frequency of each word corresponds to font size: the bigger the font, the more popular the word was.

As *Figure 3a* suggests, in the Russian tweets there were three leading words: “*people — bureaucrats — children*.” The second most frequent group was “*job — Russia — country*.” The third was “*years — rubles — month*.” It is important to note that this list of words echoed the attitudes revealed above: *money* was most often associated with people, civil servants, and *expenses* for bringing up children, while *incomes* were related to jobs and to the general situation in the country; the interrelation between *time* and money that has been discussed above is also visible.

In the Japanese sample, the 50 leading words were distributed differently (*Figure 3c*). The top position was occupied by the following three words: “*time — day — month;*” the second level of frequency by the group “*subject — direction — advantage;*” and the third one “*all — question — year*.” Thus, for the Japanese users, money was primarily associated with “time;” then came “subject,” “direction” of activity, and the “advantages” that money can give. The third position was represented by connections with the social determination of the problem (all), with questions related to the function of money, and with such categories as “year.” Here it is important to highlight the frequency of the calendar periods’ distribution: the distribution ranged from a smaller period of time to a larger one; money was regarded as a common value, daily, monthly, and yearly. Such distribution gives an idea of how the Japanese regard money matters: they move from the urgent tasks to the more distant ones, or, at any rate, current affairs have preference over future ones.

If we consider the verbs the tweeters used to represent money-related activities, it becomes evident that the most popular items denoting the Russians’ attitude to money were “pay,” “spend,” and “buy,” while the Japanese showed a different set of emphases: “do,” “finish,” and “be” (*Figure 3b*). Continuing the comparative analysis, we find out that in the Russian sample there were words that were absent in the Japanese one (*3d*), namely, “collect,” “distribute,” “steal,” “provide,” “borrow,” “seize,” etc.). Correspondingly, there were words that were not found in the Russians’ messages but were used by the Japanese sample (“be responsible for,” “weigh,” “hope,” “augment,” “multiply,” “lower,” “control,” “depend,” etc.). In the totality of the Russian words there were also less frequent ones that have not been included in the top 50: “put together,” “launder,” “embezzle,” “racket,” “gamble,” “beg,” etc. In both samples there were words like “earn,” “work,” “pay back,” etc.

Another category of words contained items that the Twitter users from both countries used but with a notable difference in frequency. Thus, words denoting various kinds of money-related activities made up the top 10 in the Japanese sample (“augment,” “lower,” “multiply,” “spread,” “weigh,” etc.), while in the Russians’ messages, they were also used (“invent,” “create,” “augment,” “manage,” etc.) but were not even in the top 100.

Quite specific differences also emerged from the messages of Japanese users, whose interests and view of life do not match the substantial features of the Russian mentality. Below are three examples of how the Japanese tackled certain problems that found little or no correspondence in the discussions between the Russian users:

- “Will cod roe *pay off*?” — the Russians did not discuss cost prices in their messages;
- “The pandemic makes many people *lose* money” — in the Russians’ messages, it was not common to discuss the correlation between the pandemic and lower standards of living; and
- “The fact that he lied to another person for his benefit does not mean he is a bad man; it is the *country* in which one can easily deceive people that is bad” — this way of reasoning about the country’s government system was not found in the Russians’ messages.

## Conclusion

1. The informational-analytical study of Internet texts according to their psychological content — a kind of study which cannot be performed in the individual, “manual” way due to the huge volume of material — is becoming more and more *popular* with researchers who use program products to conduct automatic analysis and provide psychological interpretation of the information obtained.
2. The statements that reveal Russians’ characteristic attitudes towards money can be grouped into eight main categories (people, time, country, expenses, economy, philosophic speculations, power, and incomes). These statements include both individually (buy, earn, spend, etc.) and socially oriented positions (help, borrow, put together, etc.)
3. The main economic operations with money, in the Russian Twitter users’ views, were centered on expenses and incomes that came from *paid jobs*; they gave little or no attention to entrepreneurial activities. The economic operations that were long-term or that implied accumulating money (savings and investments) were not among the topics most widely discussed; the users seemed not to be profoundly interested in them.
4. The structure of the Russians’ incomes throughout all the texts we studied was represented by such references as “job,” “salary,” and “payout,” which led us to conclude that paid jobs and social disbursements from the state were the *main* (most common) sources of income for this Russian population. Similarly, when the Russian users discussed and planned their future incomes, the 50 most common verbs denoting current and prospective activity had little to do with technologies and ways of enrichment outside paid jobs (for example, business, housekeeping, creative work, etc.) These results show the entrepreneurial inactivity of the users and the prevalence of expecting financial support from the state.
5. The word frequency analysis we performed revealed that the Russians’ main expenses were concentrated on the financial support of their families and solving their current financial problems; a rare — if not the only — case of spending money not connected with everyday life expenditures was putting aside a certain amount of money supplies for family *holidays*.
6. A comparative analysis of word frequency between the Russian and Japanese tweets, confirmed the existence of very specific features typical of these two ethnic groups o; the differences concerned both incomes/ expenses structure and patterns of financial planning. The differences also became evident when money-related economic issues were discussed. These results clearly denote that there are *important typological* features that are probably determined by the mental characteristics of different ethnic groups; these characteristics, in their turn, are determined by centuries-long cultural and historical processes in the countries in question.
7. When it comes to attitudes toward money, it became clear that there were a number of distinctive features shared by the majority of users from Russia and Japan, which were revealed through comparative analysis of their messages. This implies that it is necessary to perform detailed economic-psychological research on the attitude toward money that members of these ethno-cultural communities hold, and to find out which psychological factors determine the differences. This can be regarded as a *worthwhile objective* for further research.

## Final considerations

The present paper is illustrative of how research based on automatized analysis of texts for psychological science purposes is being carried out today. Among other things, it demonstrates the main methodological approaches currently being applied for such purposes, such as frequency analysis of words and word-combinations, assessment of the emotional background of short informal texts, bibliographic analysis of scientific literature, and scientometric analysis of trends in psychological studies. As a result, one can conclude that studying “digital traces” for psychological science purposes, despite all reservations, could be characterized as a “paradigm shift” in the empirical study of social phenomena. Although using program tools and outcomes of automatic analysis to describe psychological regularities in people’s behavior and activity is not yet suitable for creating a coherent picture of modern people’s social life, nonetheless it is a good way to assess numerous psychological elements of human life in an adequate way.

Within this type of study, it seems most important to uncover regular correlations between the overall psychological characteristics of a population and the existing social-economic state in which the society finds itself. The elaboration of this study trend is connected with the fact that “the long-lasting evolution of production, and the breakthrough development of technologies and techniques make it necessary to adjust the objectives of civilizational development, and thus human beings come to the fore, both as factors and as objectives of further social development” (Dobrokhleb, 2018, p. 144).

## Method limitations

While assessing the use of information methods for analyzing psychological phenomena, one can note a number of advantages: the speed of scientific research grows; the information base widens; scientific production becomes cheaper; accumulations of digital information are used more and more efficiently; conditions are created for studying general phenomena relevant to the society as a whole; and there is the opportunity to develop systems of monitoring the social-psychological climate in small social groups, in the workplace, and in society generally.

Along with the advantages that “digital” research technologies give, one should also mention their drawbacks: the need for innovative systems which will allow us to analyze and standardize primary data; the lack of specialists with interdisciplinary competences; the difficulties in dealing with the conceptual and terminological framework in the situation of cultural diversity; and so forth. Among the particular problems related to automatized text analysis, one can mention: the fact that certain words, concepts, and terms have a range of synonyms; the interpretation difficulties that arise when even full recognition of all the elements of a certain lexical item cannot guarantee its correct interpretation outside the context of the message; the information limits that a given library or archive has; the limited foreign language competences of the specialists; the limits that the research base has due to its being rooted in a particular language environment; and so forth.

In spite of all the drawbacks, it seems evident that the theoretical and methodological approaches and methods of this kind of research will continue to evolve, thus creating the necessary conditions for obtaining new scientific results.

## Footnotes

1 Mentality is regarded here as a way of thinking typical of a certain ethnic or other social group. This way of thinking manifests itself in emotional, value-related, and intellectual features of an ethnic group’s view of the world and distinguishes it from other communities (Kitova, & Kitov, 2020).

2 The specific feature of analysis applying grounded theory is that the theories are elaborated on the basis of quality data chosen for the purpose, and not on the basis of the whole bulk of empirical material.
